# Effect of Platelet Transfusions on Extracorporeal Life Support Oxygenator’s Function

**DOI:** 10.3389/fped.2022.826477

**Published:** 2022-03-07

**Authors:** Madhuradhar Chegondi, Niranjan Vijayakumar, Aditya Badheka, Oliver Karam

**Affiliations:** ^1^Division of Critical Care Medicine, Stead Family Children’s Hospital, Iowa City, IA, United States; ^2^Department of Pediatrics, Carver College of Medicine, University of Iowa, Iowa City, IA, United States; ^3^Division of Pediatric Critical Care Medicine, Children’s Hospital of Richmond, Richmond, VA, United States

**Keywords:** platelet transfusion, thrombosis, children, extracorporeal membrane oxygenation, hemostasis

## Abstract

**Objective:**

Bleeding is a common complication of extracorporeal membrane oxygenation (ECMO), leading to increased mortality. Since one of its main complications is bleeding, platelet transfusions are frequently prescribed for children on ECMO. However, there is currently very little information on the effect of platelet transfusions on the function of the ECMO oxygenator. Our objective was to describe the effect of platelet transfusions on oxygenator function.

**Methods:**

In this retrospective study, we included all children (<18 years) who received ECMO support in our pediatric intensive care unit (PICU) between January 2017 and December 2019. Oxygenator function, measured before and after platelet transfusion, was assessed by post-oxygenator P_*ECMO*_O_2_ and the gradient in pre- post-oxygenator pressures (Delta Pressure).

**Results:**

Over 3 years, we analyzed 235 platelet transfusions from 55 children who received ECMO support. Thirty-two (80%) of children were on veno-arterial ECMO and majority of them were peripherally cannulated. When looking at all transfusions, the post-transfusion change in delta-pressure was 0.1 mmHg (*p* = 0.69) and post-membrane P_*ECMO*_O_2_ was 6 mmHg (*p* = 0.49). However, in the subgroup with the lowest quartile of pre-transfusion oxygenator function, the post-transfusion change in delta-pressure was −5.2 ± 2.7 mmHg (*p* < 0.001) and the post-transfusion change in P_*ECMO*_O_2_ was −118 ± 49 (*p* < 0.001). The area under the ROC curve for the pre-transfusion delta-pressure and P_*ECMO*_O_2_ to predict a worsening of the oxygenator function were 0.72 (95%CI 0.63–0.81) and 0.71 (95%CI 0.64–0.78), respectively. Using regression models, pre-transfusion delta-pressure and P_*ECMO*_O_2_ were the only independent factors associated with oxygenator function worsening (*p* < 0.001).

**Conclusion:**

Our study suggests that overall, platelet transfusions do not seem to impact the ECMO oxygenator’s function. However, in the subgroup of patients with the lowest pre-transfusion oxygenator function, platelet transfusions were independently associated with a worsening function. Future studies should investigate if this warrants adjustments of the anticoagulation strategy around the platelet transfusion, especially among patients with lower oxygenator function.

## Introduction

Extracorporeal membrane oxygenation (ECMO) is a temporary support measure used in children with refractory cardiorespiratory failure. However, bleeding and thrombosis remained frequent complications. Dalton et al. reported bleeding events in 70% of children on ECMO and thrombotic events in 38% (1). These adverse events were independently associated with worse morbidity and mortality, with an overall mortality rate of 45%.

On ECMO, blood exposure to the artificial surface of the circuit, inflammatory response, and shear stress-induced platelet activation are the main reasons for a consumptive coagulopathy, which is believed to play an important role in the increased risk of thrombosis and bleeding (2, 3). Qualitative and quantitative platelet dysfunction is well described in patients receiving ECMO support, which leads clinicians to transfuse platelet and other blood product transfusion to achieve hemostatic balance (3). Among the children receiving ECMO support, 97% received at least one platelet transfusion (4). Most platelet transfusions are prophylactic in response to thrombocytopenia and children without bleeding (5). However, the optimal transfusion threshold is not well defined, and current practice is based on expert opinion (6). In addition, platelet transfusions are independently associated with increased risk of thrombosis and mortality, but surprisingly, also bleeding (4).

Some underlying mechanisms that could explain worse clinical outcomes after platelet transfusions have been explored. The activated platelets during the ECMO support seem to result in excessive circuit clot burden, especially in the oxygenator membrane (7), which in turn could increase blood flow resistance and impair the gas exchange (8). Platelet transfusions are independently associated with overall thrombotic events (4), and circuit thrombotic events (including the oxygenator) are twice more common than patient-related events (1). However, the effects of platelet transfusion on ECMO oxygenator membrane function have not yet been described. In our study, we aim to assess the impact of platelet transfusion on ECMO oxygenator functions.

## Materials and Methods

This retrospective study was conducted at a quaternary care university hospital. Following our institutional review board approval (University of Iowa IRB-01, approval number 201907785), the data was extracted from electronic medical records. All children 0–18 years of age admitted to our pediatric intensive care unit (PICU) between January 2017 and December 2019 were eligible. We included all children who received ECMO support, irrespective of the indication. We did not include patients undergoing ECMO in other units, such as the neonatal intensive care unit or the adult intensive cares. We identified ECMO procedure codes in the EMR using the International Classification of Diseases Tenth Revision Clinical Modification (ICD10-CM) code 5A15223.

### Extracorporeal Membrane Oxygenation System and Anticoagulation Policy

Extracorporeal membrane oxygenation system used during the study period remained consistent with Quadrox-D oxygenator, Rotaflow centrifugal pump (Macquet Cardiopulmonary AG, Hirrlingen, Germany), and no bladder or reservoir. An oxygen air blender was used for gas exchange across the oxygen membrane. The oxygenator heat exchanger was set at 37°C. The anticoagulation practices also remained consistent during the study period. The institutional anticoagulation protocol was followed with unfractionated heparin as a primary anticoagulant. Coagulation status was monitored every 4–8 h. The goal heparin assay (anti-Xa) was 0.3–0.7 U/mL and the aPTT goal was 1.5–2 times the control value in seconds. If there was a discrepancy between the aPTT and anti-Xa, we follow the anti-Xa level. In addition, Thromboelastogram (TEG) was performed at least daily. Per our institution ECMO policy, we transfuse platelets if the platelet count is less than 100 × 10^9/^L or irrespective of platelet count if the patient is with clinical bleeding.

### Data Collection

We collected demographic, clinical, laboratory, platelet transfusion, and ECMO circuit variables. We measured the bleeding and thrombotic event as reported in the EMR. Bleeding was defined as per extracorporeal membrane oxygenation support (ELSO) guidelines based on the red blood cell transfusion or other intervention such as surgical or endoscopic intervention (9). Thrombotic events were defined as partial of complete occlusion of a deep vein, irrespective of the site. The platelet transfusion variables included pre and post-transfusion platelet count, platelet transfusion volume, transfusion duration, concurrent other blood products, or crystalloid/colloid fluid transfusion through the ECMO circuit. The ECMO circuit variables (pre- and post-platelet transfusion) included size and site of cannulation, type of pump, type of oxygenator, type of ECMO, ECMO flow rate, RPM, duration of ECMO support at the time of platelet transfusion, and additional circuits (dialysis or plasmapheresis). Finally, we also measured all-cause mortality (at the end of the ECMO run, at PICU and hospital discharge, and at 28 and 90 days).

The primary outcome was the change in oxygenator function, as evaluated by the change in oxygenator membrane pressure gradient (delta-pressure), using the difference between the pre- and post-oxygenator pressures. The secondary outcome was the pre- post-transfusion change in post-oxygenator oxygen partial pressure (P_*ECMO*_O_2_).

To address small numerical changes, we also provided proportional changes in delta-pressure and P_*ECMO*_O_2_ as the post-transfusion change divided by the pre-transfusion value, in percentage.

To identify factors associated with worse post-oxygenation oxygenator function, we categorized the platelet transfusion events according to the 75th percentile of the change in delta-pressure (≤2 mmHg vs. >2 mmHg) and change in P_*ECMO*_O_2_ (≤ −50 mmHg vs. > −50 mmHg).

### Statistics

The categorical variables were reported as counts (*n*) and percentages; continuous data were reported as median and interquartile ranges (IQR). We used the paired Student *T*-test to evaluate the significance of P_*ECMO*_O_2_ and Delta-Pressure changes, before and after platelet transfusion, since the distribution was normal. We used Wilcoxon Rank Test to evaluate differences among groups, as the distributions were not normal. Multivariable linear regression models were developed to evaluate the post-transfusion change in P_*ECMO*_O_2_ and Delta-Pressure, as well as the cumulative effect of transfusions. Variables were considered potential predictors if they were associated with the outcome in univariate analysis (*p* < 0.10). The final model was selected using a bidirectional step-wise selection on the potential predictors with a significance criterion of the *p*-value of <0.05 to enter in the model and a *p*-value of >0.1 to be removed. No variables other than the P_*ECMO*_O_2_ and Delta-Pressure were forced into the models. We evaluated the accuracy to predict a worsening of the oxygenator function with an area under the receiver operating characteristic (AUC). AUC was considered excellent if >0.90; good between 0.80 and 0.90; fair between 0.70 and 0.79; poor between 0.60 and 0.69; and not discriminatory <0.60 (10). We identified optimal cutoff values using Youden’s J statistic. A *p*-value less than 0.05 was considered significant. All tests were 2-sided. All statistical analyses were performed with SPSS version 27 for Mac (SPSS, Chicago, IL, United States).

## Results

Over 3 years, 55 children received ECMO support. Out of them, 40 children (72.7%) received at least one platelet transfusion, for a total of 235 transfusions. Of these, 22 (55%) were males, with the median age of 4 months (IQR 0.45–33). As shown in Table 1, most children had an underlying congenital heart disease, and cardiogenic shock was the most common indication for the ECMO support. Thirty-two (80%) of children were on VA ECMO and majority of them were peripherally cannulated. Other ECMO variables, anticoagulation details and laboratory details were summarized in Table 1. Patient-related thrombotic events in the form of deep vein thrombosis were noted in 10% patients and 15% had circuit-related events requiring circuit change. The survival rate at the end of ECMO run was 67.5% (27/40) and overall 90-day survival rate was 52.5% (21/40).

**TABLE 1:** Demographic, baseline data.

Variable	All Children
Number of children	40
**Gender, *n* (%)**	
Male	22 (55)
Female	18 (45)
Age in months (median, IQR)	4 (0.45–33)
Weight in kg (median, IQR)	4.7 (3.3–16.5)
**Primary diagnosis, *n* (%)**	
Congenital heart disease	26 (65)
Respiratory	7 (17.5)
Malignancy	3 (7.5)
Sepsis	4 (10)
**ECMO indication, *n* (%)**	
Cardiogenic shock	26 (65)
Acute respiratory failure	10 (25)
Septic shock	4 (10)
**Type of ECMO, *n* (%)**	
VA	32 (80)
VV	8 (20)
**Type of ECMO cannulation, *n* (%)**	
Peripheral	24 (60)
Central	16 (40)
**ECMO priming solution, *n* (%)**	
Whole blood	26 (65)
Saline	14 (35)
**Anticoagulation use, *n* (%)**	
Yes	31 (77.5)
No	9 (22.5)
ECMO with CRRT, *n* (%)	23 (57.5)
ECMO circuit change, *n* (%)	6 (15)
Patients with clinical bleeding, *n* (%)	12 (30)
Patients with DVT, *n* (%)	4 (10)
**Laboratory parameters at ECMO initiation, median (IQR)**	
Hemoglobin (g/dL)	12.2 (9.9–14.1)
Fibrinogen (mg/dL)	234 (148–304)
Antithrombin(%)	67.4 (53.5–96.2)
Anti Xa assay (IU/mL)	0.45 (0.29–0.57)
pH	7.41 (7.36–7.45)
Lactate (mmol/L)	2.3 (1.4–4.8)
ECMO survival, *n* (%)	27 (67.5)
90-day survival	21 (52.5)

*IQR, inter quartile range; ECMO, extracorporeal membrane oxygenation; VA, venoarterial; VV, venovenous; CRRT, continuous renal replacement therapy; and DVT, deep vein thrombosis.*

As presented in Table 2, the median pre-transfusion platelet count was 101 × 10^9/^L (IQR 69-155). The median transfused platelet volume was 10 mL/kg (IQR 10-10).

**TABLE 2:** Platelet transfusion and extracorporeal membrane oxygenation (ECMO) oxygenator variables (*n* = 236 platelet transfusions).

Variables	Values
Pre-transfusion platelet count (X 10^9/^L, median and IQR)	104 (69–155)
PT volume (mL/kg, median and IQR)	10 (10–10)
PT duration (minutes, median and IQR)	60 (60–60)
Days of ECMO support prior to PT (median and IQR)	7 (3–20)
**ECMO oxygenator delta pressure (mmHg, mean + SD)**	
Pre-transfusion	14.6 ±9.3
Post-transfusion	14.8 ±9.5
**ECMO oxygenator pre-membrane PO_2_ (mmHg, mean + SD)**	
Pre-transfusion	123 ±82
Post-transfusion	121 +86
**ECMO oxygenator post-membrane PO_2_ (mmHg, mean + SD)**	
Pre-transfusion	372 ±127
Post-transfusion	366 ±132

*IQR, inter quartile range; PT, platelet transfusion; ECMO, extracorporeal membrane oxygenation; and PO2, partial pressure of oxygen.*

**FIGURE 1:**
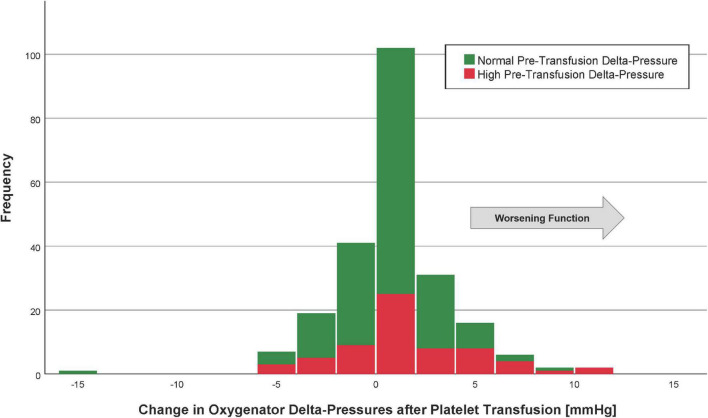
Histogram of the change in oxygenator delta-pressures after platelet transfusion. Patients with the highest quartile of pre-transfusion oxygenator delta-pressures are in red (“high” delta-pressures), whereas those with oxygenator delta-pressures lowest three quartiles are in green (“normal” delta-pressures). The arrow indicates the direction of worsening oxygenator function.

The mean oxygenator delta-pressures were 14.6 ± 9.3 mmHg (before platelet transfusion) and 14.8 ± 9.5 mmHg (after platelet transfusion). The median change in delta-pressure was 0 mmHg (IQR −1 to 2) mmHg, which was not a statistically significant difference from pre-transfusion baseline (*p* = 0.69, Figure 1). The average proportional change was −5%. In a linear regression model, the only factor associated with a change in delta-pressures >2 mmHg (i.e., worsening post-transfusion oxygenator function based on the 75th percentile) was the pre-transfusion delta-pressure (*p* < 0.001, Table 3).

**TABLE 3:** Laboratory values and ECMO variables according to change in Delta-Pressure > 2 mmHg* (*n* = 232 platelet transfusions).

Variables	Change in Delta-Pressure ≤ 2 mmHg after platelet transfusion *n* = 197	Change in Delta-Pressure > 2 mmHg after platelet transfusion *n* = 35	*P*-value
Hemoglobin	11.1 (9.8–12.9)	11.3 (9.2–13.2)	0.93
Pre-transfusion Platelet Count	98 (66–137)	104 (80–134)	0.28
Anti-Xa	0.30 (0.13–0.50)	0.23 (0.10–0.51)	0.27
Antithrombin-	85 (67–108)	94 (71–106)	0.49
Fibrinogen	265 (184–386)	244 (190–386)	0.95
Number of platelet transfusions	4 (2–8)	5 (3–7)	0.55
ECMO Flow Rate	0.83 (0.44–1.75)	1.01 (0.57–1.44)	0.24
Pre-transfusion Delta-Pressure	11 (9–16)	17 (12–25)	**<0.001**
Pre-transfusion P_*ECMO*_O_2_	411 (271–481)	401 (235–500)	0.80

Values are presented as medians and interquartile range. ECMO, Extracorporeal membrane oxygenation. *A change in Delta-Pressure of 2 mmHg was the 75th percentile of lowest pre-transfusion oxygenator function. The bold p values are those that are statistically significant.

**FIGURE 2:**
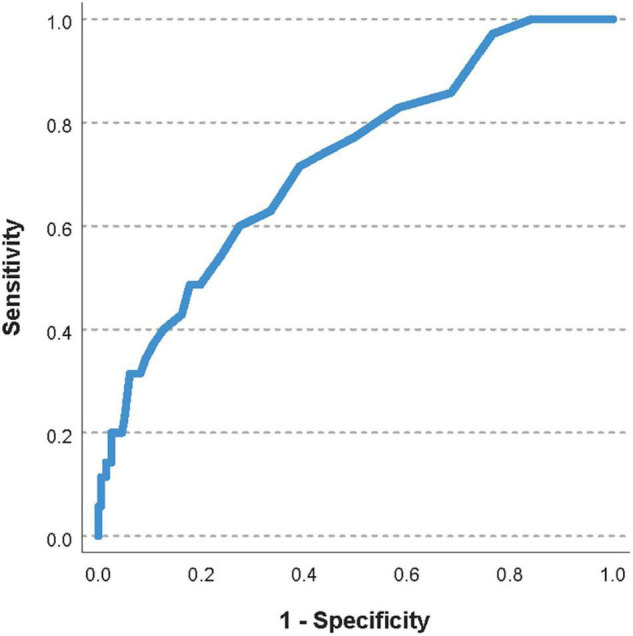
Receiver operating characteristic (ROC) curve of the pre-transfusion delta-pressure to predict a worsening of the oxygenator function. The area under the ROC curve was 0.72 (95%CI 0.63–0.81).

The area under the ROC curve for the pre-transfusion delta-pressure to predict a worsening of the oxygenator function was 0.72 (95%CI 0.63–0.81, Figure 2). The optimal pre-transfusion delta-pressure cutoff to predict worsening oxygenator function, identified by Youden’s J statistic, was 16 mmHg. Patients with a pre-transfusion delta-pressure above this cutoff (>16 mmHg) had significantly worse post-transfusion delta-pressure, when compared to patients with delta-pressure below this cutoff (proportional change in delta-pressure −9% vs. +2%, *p* < 0.04).

**FIGURE 3:**
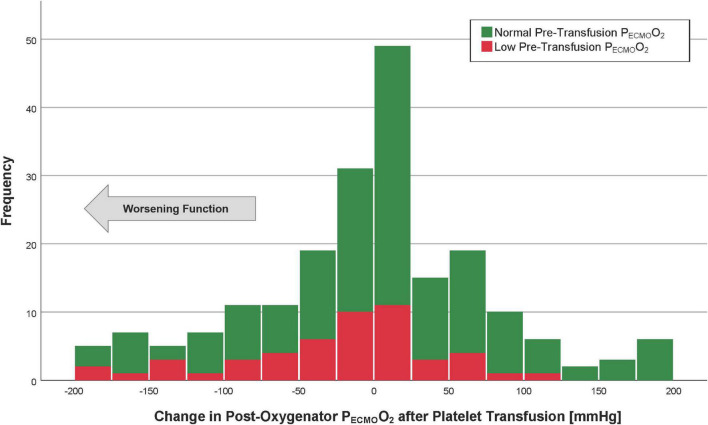
Histogram of the post-oxygenator P_*ECMO*_O_2_ after platelet transfusion. Patients with the lowest quartile of pre-transfusion post-oxygenator PO_2_ are in red (“low” P_*ECMO*_O_2_), whereas those with post-oxygenator P_*ECMO*_O_2_ highest three quartiles are in green (“normal” P_*ECMO*_O_2_). The arrow indicates the direction of worsening oxygenator function.

The mean post-membrane P_*ECMO*_O_2_ values were 370 ± 127 (before platelet transfusion) and 366 ± 132 mmHg (after platelet transfusion). The median change in post-membrane P_*ECMO*_O_2_ was 6 mmHg (IQR −45 to 50), which was not a statistically significant difference from pre-transfusion baseline (*p* = 0.49, Figure 3). The average proportional change was −2%. In a linear regression model, the only factor associated with a change in P_*ECMO*_O_2_ > −50 mmHg (i.e., worsening post-transfusion oxygenator function based on the 75th percentile) was the pre-transfusion P_*ECMO*_O_2_ (*p* < 0.001, Table 4).

**TABLE 4:** Laboratory values and ECMO variables according to change in post-oxygenator P_*ECMO*_O_2_≤−50 mmHg * (*n* = 219 platelet transfusions).

Variables	Change in P_*ECMO*_O_2_ > −50 mmHg after platelet transfusion *n* = 166	Change in P_*ECMO*_O_2_ ≤ −50 mmHg after platelet transfusion *n* = 53	*P*-value
Hemoglobin	11.2 (9.8–12.9)	11.8 (10.1–12.9)	0.40
Pre-transfusion Platelet Count	99 (69–132)	108 (79–141)	0.29
Anti-Xa	0.30 (0.10–0.50)	0.36 (0.17–0.65)	0.22
Antithrombin-	88 (67–112)	76 (68–98)	0.33
Fibrinogen	260 (183–387)	301 (212–379)	0.71
Number of platelet transfusions	4 (2–7)	3 (2–6)	0.73
ECMO Flow Rate	0.83 (0.45–1.41)	0.84 (0.41–1.69)	0.40
Pre-transfusion Delta-Pressure	13 (9–18)	12 (9–18)	0.60
Pre-transfusion P_*ECMO*_O_2_	431 (277–494)	313 (235–401)	**<0.001**

Values are presented as medians and interquartile range. ECMO, Extracorporeal membrane oxygenation. *A change in post-oxygenator P_ECMO_O_2_ of 50 mmHg was the 75th percentile of lowest pre-transfusion oxygenator function.

*The bold p values are those that are statistically significant.*

**FIGURE 4:**
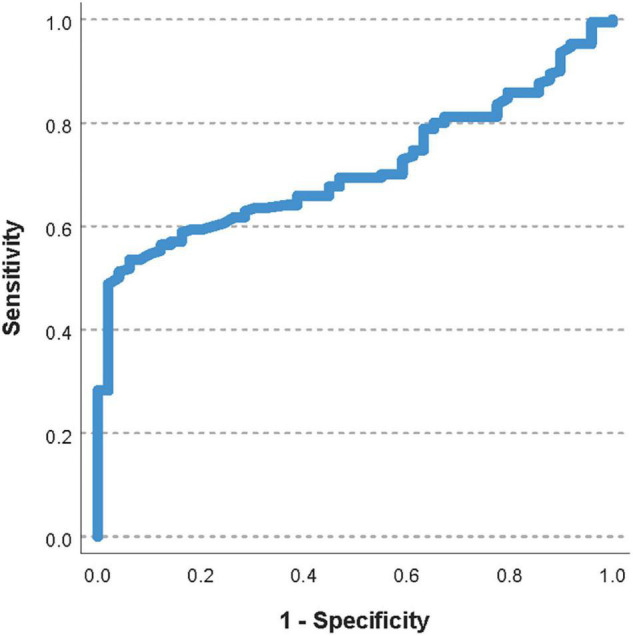
Receiver operating characteristic curve of the pre-transfusion P_*ECMO*_O_2_ to predict a worsening of the oxygenator function. The area under the ROC curve was 0.71 (95%CI 0.64–0.78).

The area under the ROC curve for the pre-transfusion P_*ECMO*_O_2_ to predict a worsening of the oxygenator function was 0.71 (95%CI 0.64–0.78, Figure 4). The optimal pre-transfusion P_*ECMO*_O_2_ cutoff to predict worsening oxygenator function, identified by Youden’s J statistic, was 216 mmHg. Patients with a pre-transfusion P_*ECMO*_O_2_ below this cutoff (<216 mmHg) had significantly worse post-transfusion P_*ECMO*_O_2_, when compared to patients with P_*ECMO*_O_2_ above this cutoff (proportional change in P_*ECMO*_O_2_ 17% vs. 0%, *p* < 0.006).

When adjusting for the pre-transfusion oxygenator function, there was no cumulative effect of the number of transfusions on post-transfusion change in delta-pressure (*p* = 0.57) or P_*ECMO*_O_2_ (*p* = 0.68).

## Discussion

In this retrospective single-center observational study, we evaluated the effect of platelet transfusion on ECMO oxygenator function, as assessed by a change in oxygenator delta-pressures or P_*ECMO*_O_2_. When looking at all platelet transfusions, our results suggest that there doesn’t seem to be a significant effect on ECMO oxygenator function. However, in the subgroup of patients with the lowest oxygenator function, platelet transfusions were independently associated with a worsening function, both in terms of change in delta-pressure and P_*ECMO*_O_2_.

Various studies have evaluated platelets on ECMO. In a prospective study, Chandler reported an increased activation and injury of platelet, red blood cell and endothelial cell in children on ECMO (11). This study objectively measured the extent of cellular injury using extracellular vesicles estimation. Another pathophysiological mechanism for thrombosis and microthrombi in the oxygenator is circuit related activation of complement and coagulation cascades following blood contact with artificial surface (11, 12). Lehle et al. studied oxygenator membranes after their removal from ECMO circuit, using electron and florescence microscopy (8). They demonstrated platelet and red blood cell accumulation, along with fibrin strands and thrombin deposition on the membrane. They also reported an increase blood flow resistance and impaired gas exchange capacity (8). Similarly, in our subgroup of patients with the lowest oxygenator function, platelet transfusions further worsening function with platelet transfusions.

In our study, 73% of our patients received platelet transfusion, while only 30% of our patients experienced a bleeding event, suggests that many platelet transfusions were prophylactic (i.e., with the intent of preventing bleeding, based on a preset transfusion threshold of 100 × 10^9^/L and/or the perceived risk of bleeding by the attending). It has been reported that immediately following ECMO support initiation, neonates drop their platelet count by 25–60% and children by 30%, due to hemodilution and platelet destruction by the pump (4, 13). The resulting thrombocytopenia during ECMO support often triggers prophylactic platelet transfusions, which in turn results in immune dysregulation and microthrombi formation leading to multiorgan dysfunction and mortality (14, 15). In children on ECMO, Cashen et al. reported that the platelet transfusion volume, but not the patient’s platelet count, was independently associated with increased thrombotic events on the following day (4). In our study, 10% had patient-related thrombotic events and 15% had circuit-related events requiring circuit change. However, given the retrospective nature of our study, we cannot extrapolate the casual relationship between platelet transfusion and circuit change.

This study’s main strength resides in the innovative hypothesis. Indeed, the effect of platelet transfusion on ECMO oxygenator function has not been previously studied. However, our study has several limitations. First it is a single-center retrospective study with small sample size. Secondly, though platelet transfusion worsens the oxygenator’s function in the subgroup of patients with lower pre-transfusion oxygenator function, we cannot infer a cause-and-effect relationship. Nonetheless, our regression models did not identify other potential cofactors. Third, we didn’t evaluate some other potential confounding factors, such as plasma transfusions or hemostatic medications. Fourth, although our results are statistically significant, the clinical relevance is unknown.

## Conclusion

Our study suggests that overall, platelet transfusions do not seem to impact the ECMO oxygenator’s function. However, in the subgroup of patients with the lowest pre-transfusion oxygenator function, platelet transfusions were independently associated with a worsening function, both in terms of change in PO2 and delta-pressure. These findings highlight the need to take the oxygenator performance into account when defining platelet transfusion thresholds as “prophylactic” transfusions in these situations could be associated with further worsening of oxygenator function. Future studies should evaluate the clinical significance of platelet transfusions on the oxygenator function and should investigate if this warrants adjustments of the anticoagulation strategy around the platelet transfusion, especially among patients with lower oxygenator function.

## Data Availability Statement

The raw data supporting the conclusions of this article will be made available by the authors, without undue reservation.

## Ethics Statement

The studies involving human participants were reviewed and approved by the Hawk IRB, University of Iowa (IRB # 201907785). Written informed consent from the participants’ legal guardian/next of kin was not required to participate in this study in accordance with the national legislation and the institutional requirements.

## Author Contributions

All authors listed have made a substantial, direct, and intellectual contribution to the work, and approved it for publication.

## Conflict of Interest

The authors declare that the research was conducted in the absence of any commercial or financial relationships that could be construed as a potential conflict of interest.

## Publisher’s Note

All claims expressed in this article are solely those of the authors and do not necessarily represent those of their affiliated organizations, or those of the publisher, the editors and the reviewers. Any product that may be evaluated in this article, or claim that may be made by its manufacturer, is not guaranteed or endorsed by the publisher.
